# Impedance Spectroscopy of Encapsulated Single Graphene Layers

**DOI:** 10.3390/nano12050804

**Published:** 2022-02-27

**Authors:** Rainer Schmidt, Félix Carrascoso Plana, Norbert Marcel Nemes, Federico Mompeán, Mar García-Hernández

**Affiliations:** 1 Campo Moncloa, Grupo de Física de Materiales Complejos (GFMC), Dpto. de Física de Materiales, Facultad de Ciencias Físicas, Universidad Complutense de Madrid, 28040 Madrid, Spain; nmnemes@fis.ucm.es; 2 Unidad Asociada “Laboratorio de Heteroestructuras con Aplicación en Spintrónica”, UCM-CSIC, Sor Juana Ines de la Cruz, 3, Cantoblanco, 28049 Madrid, Spain; 3 Instituto de Ciencia de Materiales de Madrid—Consejo Superior de Investigaciones Cientificas (ICMM-CSIC), Materials Science Factory, 2D Foundry Group, Cantoblanco, 28049 Madrid, Spain; felixcar@ucm.es (F.C.P.); federico.mompean@csic.es (F.M.); marmar@icmm.csic.es (M.G.-H.)

**Keywords:** single-layer graphene, impedance spectroscopy, electrode resistance

## Abstract

In this work, we demonstrate the use of electrical impedance spectroscopy (EIS) for the disentanglement of several dielectric contributions in encapsulated single graphene layers. The dielectric data strongly vary qualitatively with the nominal graphene resistance. In the case of sufficiently low resistance of the graphene layers, the dielectric spectra are dominated by inductive contributions, which allow for disentanglement of the electrode/graphene interface resistance from the intrinsic graphene resistance by the application of an adequate equivalent circuit model. Higher resistance of the graphene layers leads to predominantly capacitive dielectric contributions, and the deconvolution is not feasible due to the experimental high frequency limit of the EIS technique.

## 1. Introduction

Since the discovery of single-atom graphene layers in the year 2004, large research efforts have been dedicated to the investigation of their fundamental properties and more practical aspects in terms of the handling and incorporation of graphene into functional devices for potential application in the electronics industry [1,2,3,4,5,6,7,8,9,10,11].

Single-layer graphene (SLG) is not only a promising candidate as an electrode material in electrochemical applications [12], but also exhibits transparency, superior combined mechanical stability and flexibility and gives rise to several fascinating charge transport phenomena. In zero-band gap graphene, metallic or ballistic charge transport has been reported [13,14,15,16,17,18], whereas variable-range hopping has been observed in semi-conducting graphene [19]. In its pure form, SLG is predicted to be a zero-band gap semiconductor, where the valence and conduction bands touch at the Dirac points in the dispersion relation of electron energy *E* vs. propagation wave vector ***k*** [20,21]. Conversely, asymmetrical strain distributions in SLG have been shown to lead to the opening of a small band gap [22,23]. Asymmetrical strain can be caused by a small level of warping or bending of the graphene layers, as well as by impurities, where the opening band gap then leads to semiconducting charge transport with small activation energies. The electronic properties of bi-layer graphene (BLG) slightly vary, where the band gap may also be induced by the application of transverse electric fields [24,25,26].

One of the hurdles on the way to commercial integration of graphene layers into electronic devices is the precise understanding of the role of the electrode resistance, which usually arises at the interface between electrode and graphene layer. This extrinsic resistance is usually referred to as the contact or electrode resistance, where here in this work, the latter term of electrode resistance will be used consistently.

In potential applications such as graphene-based transistors [27,28,29,30,31], the electrode resistance may have a limiting effect on the device performance as manifested by low frequency 1/*f* noise in graphene devices [32]. Conversely, in other applications, such as solar cells [33,34,35], barristors [36] or sensors [37], the formation of a Schottky barrier between graphene and Si leads to an interface barrier resistance that is beneficial to the diode performance of such devices. In either case, the detection, quantification and either elimination or optimisation of the electrode or interface resistance is imperative to optimise the performance of graphene-based devices. In the case of a metallic electrode/graphene interfaces, the exact value of the electrode resistance may depend on several factors such as the choice of the electrode material [38,39], the processing parameters involved with the lithography processes that is often used to pattern the electrodes [40,41], and the shape of the electrodes [42]. For the fabrication of electrodes, there are three main possibilities: (1) deposition of electrodes onto graphene layers (top-electrodes), (2) transfer of graphene layers onto a template with pre-defined electrodes (bottom-electrodes), or probably most promising, (3) the deposition of 1D electrodes at the edges of 2D graphene layers (side electrodes) [43].

For the characterization of the electrode resistance at the electrode/graphene interfaces in terms of the graphene surface potential and work function, the use of Kelvin probe force microscopy (KPFM) and theoretical predictions by density functional theory (DFT) have been demonstrated in the literature [39,44,45,46]. However, for a precise quantitative determination and understanding of the electrode resistance, the full disentanglement of the extrinsic electrode resistance at the electrode/graphene interfaces from the two-dimensional intrinsic charge transport in graphene layers is required. Such disentanglement has been attempted previously for graphene layers contacted with Ni or other metallic top electrodes by the employment of transmission line measurements (TLMs) [40,47,48] and in the case of insulating graphene/Si Schottky diode interfaces by the use of electrical impedance spectroscopy (EIS) [49]. However, for the metallic top electrodes, the disentanglement has not been achieved so far by the use of EIS, to the best of the authors’ knowledge. Such a disentanglement is demonstrated here in this work, where EIS was applied to encapsulated graphene layer devices with a sheet resistance in the range of ≈1 kΩ/□, where the devices were contacted by Ni/Pd 1D side electrodes. A full disentanglement could be achieved here for the first time but only for devices with sufficiently low nominal graphene layer resistance (not the sheet resistance), where the former was adjusted by changing the graphene device width. The disentanglement was achieved by fitting the dielectric data to an adequate equivalent circuit model accounting for the electrical resistance of extrinsic electrode and intrinsic layer contributions separately.

For devices with higher nominal graphene resistance, the determination of the electrode/graphene interface resistance by EIS and concomitant equivalent circuit fitting of the data was not feasible. This was due to the fact that the equivalent circuit needed to be modified in terms of the constituent components (i.e., resistors, capacitors or inductors) due to such increased nominal graphene resistance, and the electrode contribution could not be fitted separately anymore. The equivalent circuits proposed here may be generally valid for EIS characterizations of graphene layers.

## 2. Electrical Impedance Spectroscopy (EIS)

The EIS technique is well established to allow for disentanglement of intrinsic and extrinsic resistances in functional materials that contribute to the overall direct current (DC) resistance. EIS has, thus, been widely applied in polycrystalline bulk materials [50,51] as well as in thin and thick films [52,53,54,55], including graphene-based devices [49].

**Figure 1:**
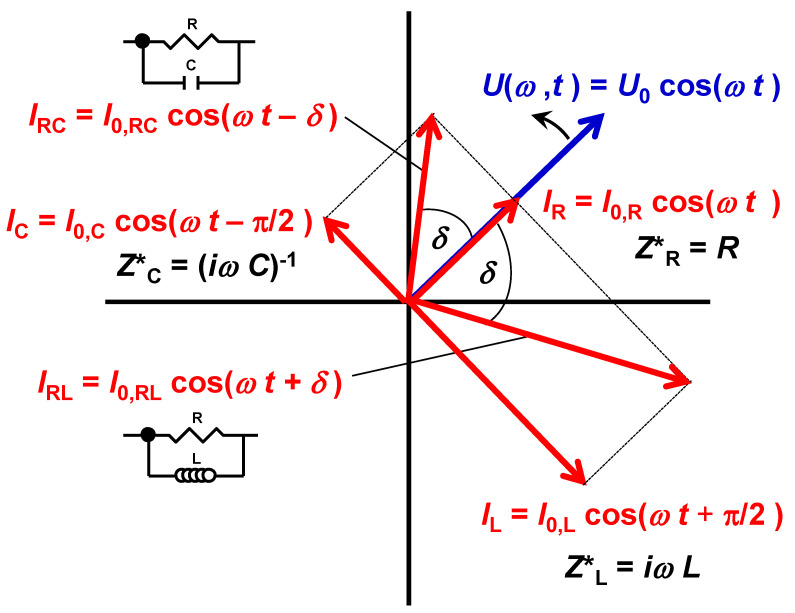
Phasor diagram for an applied voltage *U* (blue arrow) and the current response (red arrows) of an ideal resistor (*I*_R_), ideal capacitor (*I*_C_), ideal inductor (*I*_L_), ideal RC element (*I*_RC_) and ideal RL element (*I*_RL_). The complex definitions $Z_{e}^{*}$ of the impedance for *e* = R, C and L components are given. The current response of different components is given by the amplitude of the current response (length of the red arrows) and by a characteristic phase shift *δ*.

EIS experiments consist of an electric stimulus in terms of a time (*t*)-dependent alternating voltage U(*ω*, *t*) of variable angular frequency *ω* and fixed amplitude *U*_0_ applied to the sample: *U*(*ω*, *t*) = *U*_0_ cos(*ω*·*t*). Effectively, the amplitude *I*_0_ and the phase shift *δ* of the alternating current response signal *I* are measured over a wide frequency range: *I* (*ω*, *t*) = *I*_0_ cos(*ω t* + *δ*). One period of the applied voltage stimulus corresponds to a 2*π* rotation of the *U*(*ω*, *t*) arrow on the phasor diagram shown in Figure 1. The response currents of common ideal circuit elements are (1) in phase (*δ* = 0) with respect to the applied voltage for an ideal resistor (R), *I*_R_, (2) out of phase by *δ* = −*π*/2 for an ideal capacitor (C) with preceding current, *I*_C_, and (3) out of phase by *δ* = +*π*/2 for an ideal inductor (L) with lagging current, *I*_L_, where all phase angles are time independent.

To obtain a physically meaningful interpretation, the impedance needs to be defined as a time independent complex number Z* = *Z*’ + i*Z*’’, where a capacitive negative phase shift leads to a negative imaginary part of the impedance *Z*’’, and a positive phase shift leads to positive *Z*’’. The equivalent circuits applied to experimental impedance spectroscopy data are commonly made up of conventional parallel RC elements in the case of insulating and semiconducting samples with dominating capacitive contributions [50], whereas for more conducting samples with inductive dominated spectra, the equivalent circuits may be made up of less conventional RL elements [53]. Note that the phase shift measured must be either negative (capacitive) or positive (inductive). This implies that capacitive contributions (with negative *Z*’’) are not accessible for spectra dominated by inductive contributions (positive *Z*’’) and vice versa.

In the case of RC elements, the ideal capacitor is often replaced by a constant phase element (CPE), sometimes also termed a Q-element, leading to R-CPE or RQ elements. On a microscopic level, the CPE behaviour can be interpreted in terms of a broadening of the distribution of relaxation times *τ*, where *τ* = R × C [50,54].

## 3. Materials and Methods

Encapsulated graphene single layers were fabricated by transferring graphene on top of an Al_2_O_3_ film that was deposited previously onto sapphire substrates by atomic layer deposition (ALD) using tetramethylammonium hydroxide (TMAH) and water precursors [56]. The graphene was patterned into channels by defining a photoresist mask using optical lithography and sequential oxygen/argon reactive ion etching to fabricate various graphene devices with different device length *ℓ* between 2–11.2 μm. The graphene or device width *w* was fixed to *w* = 50 μm in the first more conducting sample A, whereas *w* = 10 μm was chosen in a second sample B to intentionally obtain higher nominal graphene resistances. Next, side electrodes to the graphene were sputter deposited (20 nm Ni followed by 20 nm Pd) onto a lithographically defined photoresist pattern followed by lift-off. 

**Figure 2:**
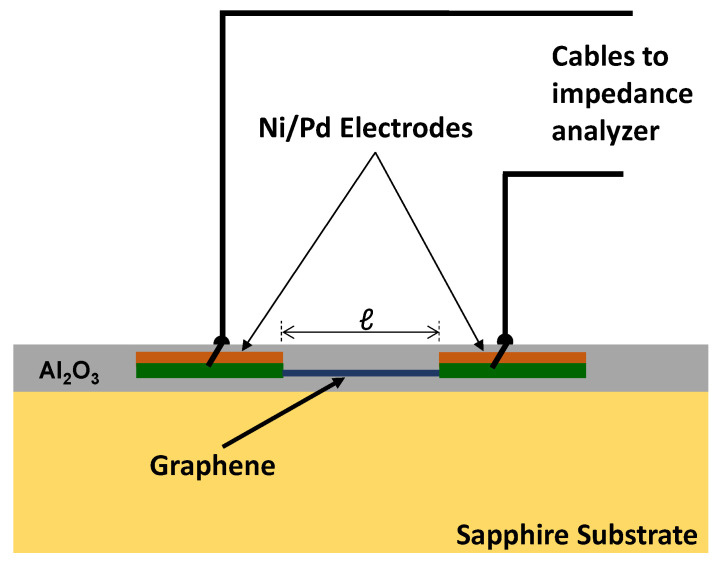
Schematic drawing of the measurement set-up for electrical impedance spectroscopy on Al_2_O_3_ encapsulated graphene layer devices with variable length *ℓ* and a fixed device width of 50 and 10 μm for samples A and B, respectively. Contact between measurement cables and the Ni/Pd electrodes was made by punching through the Al_2_O_3_ layer by wire bonding as indicated. One sample A was measured with Ag-painted top electrodes without punching through the Al_2_O_3_ layer for control purposes, which leaves an insulating Al_2_O_3_ barrier in the measurement circuit.

The device length *ℓ* is considered to be the distance between the two side electrodes, i.e., the length of the graphene layer measured (see Figure 2). The devices were finished by depositing an additional ≈50 nm Al_2_O_3_ layer on top of the full arrangement of pre-deposited Al_2_O_3_, graphene and side electrodes, again with an ALD process (TMAH and water precursors). Figure 2 shows a schematic drawing of the device architecture and the electrode configuration. Every sample contained different devices with at least 5 different device lengths *ℓ*. The two Ni/Pd measurement electrodes were contacted by wire bonding, punching across the insulating Al_2_O_3_ layers (see Figure 2). For control purposes, only for sample A, the electrical wires were attached alternatively with Ag paint (not shown). In the former case, the Al_2_O_3_ layer would be bypassed and eliminated from the circuit, whereas in the latter case, the Al_2_O_3_ layer would appear as a blocking barrier in the impedance spectra as demonstrated in the results section below. EIS was performed using a QuadTech impedance analyser and a Quantum Design PPMS measurement system. The impedance analyser was operated at variable frequencies (*f*) between 20 Hz–1 MHz with an applied voltage of 20 mV amplitude, whereas the PPMS system provided variable temperature *T* (1.7–320 K). A special sample holder was custom built (Janis Research Ltd., Woburn, MA, USA) to fit into the PPMS to minimize the internal probe capacitance (≈0.2 pF) and maximize the internal probe resistance (≈10 GΩ), which is both detrimental for reliable EIS measurements.

The sample tray at the bottom of the probe was equipped with drop-down pins with a mechanical load to ensure optimal contact to the sample electrodes. Further EIS measurements were carried out using an Alpha Analyser Novocontrol system operating at 1 Hz–10 MHz using an applied AC voltage signal of 100 mV amplitude under various *T* between 160 and 560 K upon heating. Note that the graphene impedance was always measured in an in-plane measurement configuration. 

All dielectric data were collected in terms of the real and imaginary parts (*Z*’, *Z*’’) of the complex impedance Z* = *Z*’ + i*Z*’’ under steady state conditions, where the selected *T* was allowed to settle for ≈10 min before taking impedance readings. Equivalent circuit fitting of the dielectric data was performed by using commercial Z-View^®^ software.

## 4. Results

### 4.1. Equivalent Circuit Fitting

**Figure 3:**
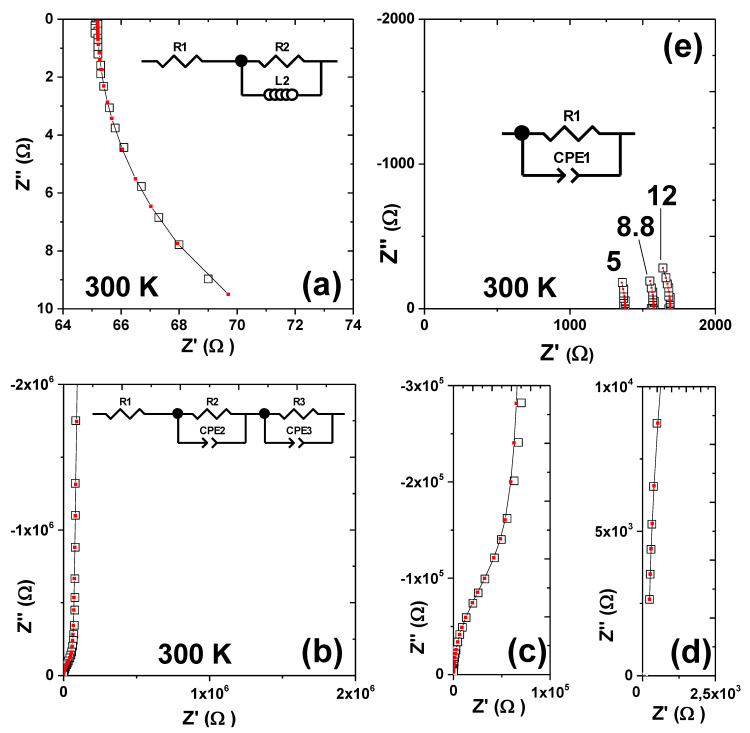
−*Z*’’ vs. *Z*’ complex impedance plane plots taken at 300 K for different samples. Open squares represent the data; red squares and solid lines represent equivalent circuit fits. The circuit models used are shown in the figure insets. (**a**) Inductive contributions are dominating for the case of the more conducting sample A, indicated by positive *Z*’’. The positive *Z*’’ is plotted as a negative value on the −*Z*’’ vs. *Z*’ (i.e., the semicircle points downwards), which is the more common way to plot impedance data. (**b**–**d**) Different magnifications of −*Z*’’ vs. *Z*’ plots for the same sample A, but with Ag painted electrodes leaving an insulating Al_2_O_3_ dielectric contribution in the circuit. (**e**) Capacitive contributions are dominating for the case of the less conducting sample B measured without the Al_2_O_3_ in the circuit, indicated by negative *Z*’’. Three different devices on the same sample were measured with 5, 8.8 and 12 μm device length *ℓ* as indicated.

As mentioned in Section 2, inductive contributions to the impedance are reflected by a positive phase shift leading to positive imaginary parts of the impedance *Z*’’, whereas capacitive behaviour leads to a negative phase shift and negative *Z*’’. Figure 3a displays a −*Z*’’ vs *Z*’ (or Nyquist) plot obtained from the graphene devices with a larger device width of 50 μm on the first more conducting sample A. In this case, a positive *Z*’’ at all frequencies is detected, which can be modelled with an equivalent circuit containing a parallel RL element and a single resistor in series as indicated in the Figure 3a inset. Note that the expected semicircle is only partially visible due to the high-*f* limit of the impedance analyser. Further note that capacitive contributions to the impedance would still be present and are expected to have a perceptible effect on the data. However, their contribution was not strong enough to allow for fitting with an equivalent circuit model containing an additional capacitor. The small discrepancy between the model and data shown in Figure 3a may be explained though by small capacitive contributions. It will be argued below in Section 4.2 that R1 represents the intrinsic graphene resistance, because it scales with the device length *ℓ*. Conversely, R2 is approximately constant and does not scale with *ℓ*. R2 may therefore be interpreted as an extrinsic contribution that arises from electrode interfaces, electrodes, cables or any other contributions that do not scale with *ℓ*. Generally, a good agreement between data and fitted curves is observed. 

In Figure 3b, negative *Z*’’ due to dominating capacitive contributions and a large pike in form of an approximately perpendicular curve are displayed for the data taken from the same sample A but with painted electrodes with an insulating Al_2_O_3_ layer in the circuit, for control purposes. The perpendicular curve can be interpreted as the onset of a large semicircle that cannot be resolved leading to the capacitive behaviour reflected by negative *Z*’’. The diameter of a capacitive semicircle that typically appears in −*Z*’’ vs. *Z*’ (or Nyquist) plots with negative *Z*’’ corresponds to the resistance of the respective RC element. Therefore, the onset of a massive conventional semicircle can be interpreted as an indication of an electrically insulating contribution, which may well be associated with the Al_2_O_3_ layer. Note that this contribution is absent in Figure 3a, where contact was made to the Ni/Pd electrodes by punching through the Al_2_O_3_ layer. At intermediate *f*, Figure 3b shows the indications of an overlapping semicircle of drastically smaller dimensions. This is demonstrated more clearly in Figure 3c, which shows a magnification of the −*Z*’’ vs. *Z*’ plots at intermediate *f*-range. Close inspection of the −*Z*’’ vs. *Z*’ plots at the high *f*-range (Figure 3d) reveals that the −*Z*’’ vs. *Z*’ curves may not pass through the origin of the plot. This apparent non-zero intercept with the real *Z*’ axis is indicative of a single resistor in the circuit [54]. Therefore, the data shown in Figure 3b–d can be modelled with a single resistor R1 and two conventional RC elements in series, where the ideal capacitors had been replaced by CPEs or Q elements. The full equivalent circuit model is displayed in the inset of Figure 3b. It will be argued below that R1 again represents the intrinsic graphene resistance, because R1 scales with the device length *ℓ*, despite the presence of the insulating Al_2_O_3_ layer. This was the expected control result, consistent with the previous sample.

Conversely, R2 and R3 do not scale with *ℓ*. R2-CPE2 may be associated with an interface contribution possibly between Al_2_O_3_ and the Ag electrodes, whereas R3-CPE3 can be associated with the charge blocking Al_2_O_3_ layer. Note that the resistance R3 had to be set to infinity for a valid equivalent circuit fit, which confirms the charge blocking behaviour of the insulating Al_2_O_3_ layer. Note further that the painted top Ag electrodes had irregular shape and the geometrical factors and specific capacitance values were not accessible. Therefore, the nominal capacitance values from CPE3 and CPE2 in the range of 10–100 pF are not meaningful. The CPE exponents were both in the range of 0.99–1, indicating almost ideal dielectric behaviour as expected for dielectric contributions from thin interfaces.

Figure 3e shows EIS data from the second sample B, which was fabricated with a smaller device width (10 μm) to intentionally increase the graphene resistance. Note that contact was made by punching across the insulating Al_2_O_3_ layer using wire bonding to eliminate it from the circuit. The onset of a conventional semicircle with negative *Z*’’ as a manifestation of dominating capacitive contributions is indicated, although contact was made to the graphene layers by punching through the insulating Al_2_O_3_. This implies that the decrease in the device width from 50 to 10 μm had a distinct qualitative effect on the resulting impedance spectra, i.e., changing the spectra from being dominated by inductive to capacitive behaviour. A possible explanation for this may be a significant reduction of the inductance of the graphene layer here. Figure 3e shows that the disentanglement of different contributions is not possible because the data can be fitted with only one R-CPE or RQ element The model is shown in the Figure 3e inset. The single R-CPE or RQ contribution does not exclude the possibility that two semicircles or a non-zero x-axis intercept may well exist and are expected, since multiple dielectric contributions are likely to be present. It is simply the high-*f* resolution limit of 1 MHz that impedes access to higher *f*-ranges, and the possible existence of high-*f* contributions cannot be tested. In the next section, it is argued that R1 represents the entire device resistance as a sum of all different contributions, because R1 changes with the device length *ℓ*, but an additional residual resistance seems to be present. These findings imply that neither the intrinsic graphene resistance nor the sheet resistance could be determined in sample B.

Generally, all equivalent circuit models shown in Figure 3 had been chosen under the strict selection criteria that the correct model must be physically meaningful, not be overdetermined, and must fit the data satisfactorily with low fitting errors (< 5%) for each circuit element.

### 4.2. Resistance Scaling

**Figure 4:**
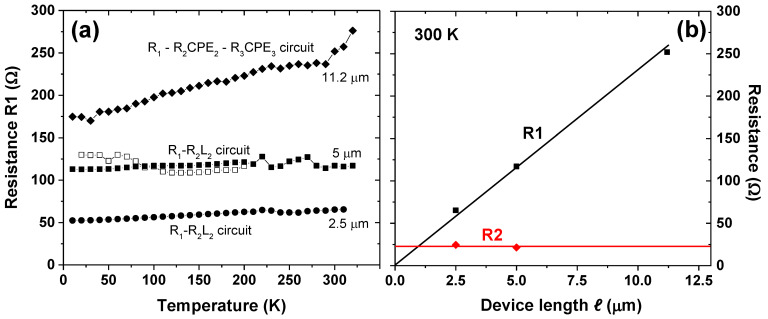
(**a**) *T*-dependence of the graphene resistance R1 in sample A, extracted from the models in Figure 3a,b for devices with different length *ℓ*. (**b**) Resistance R1 at 300 K and the resistance R2 from the model in Figure 3a. R1 scales with the device length *ℓ*, R2 is independent of the device length. R1 may thus represent the intrinsic graphene resistance and R2 extrinsic contributions.

To associate different equivalent circuit components with certain areas in the sample, it is helpful to analyse their trends of the resistance with the device length *ℓ*. Figure 4a shows the resistance R1 obtained from graphene devices with different *ℓ* from sample A (*w* = 50 μm). R1 had been extracted from the models shown in Figure 3a,b and was plotted vs. *T*. A continuous increase in resistance with *T* is observed, indicating metallic type charge transport that may be associated with the intrinsic graphene resistance. The association of R1 with an intrinsic graphene contribution is justified in Figure 4b, where the resistance R1 is plotted vs. the device length *ℓ*. R1 approximately scales with *ℓ*, where the linear trend line may pass through or close to zero. The sheet resistances for the devices with *ℓ* = 2.5, 5 and 11.2 μm are, therefore, similar and were calculated with a rough approximation to be 1, 1.15, and 0.93 kΩ/□, respectively. Note that these values for encapsulated graphene are slightly lower than the 1.84 kΩ/□ reported for free standing graphene [57], and the 1.48 kΩ/□ obtained from a rough estimate from early theoretical calculations on graphite [58]. For approximating the sheet resistance, the *T*-dependence of R1 had been averaged out. Conversely, the second resistor R2 from the model shown in Figure 3a shows approximately constant resistance and no tendency with *ℓ* and, thus, may be associated with extrinsic contributions from the electrode resistance and the measurement cables (Figure 4b). Thus, it may be concluded that the equivalent circuit in Figure 3a may well be suitable to disentangle the intrinsic graphene resistance R1 from extrinsic contributions represented by R2. The inductance L2 was found to be *T*-independent in the range of 2–2.5 mH, with only small changes with the device length *ℓ* (data not shown).

**Figure 5:**
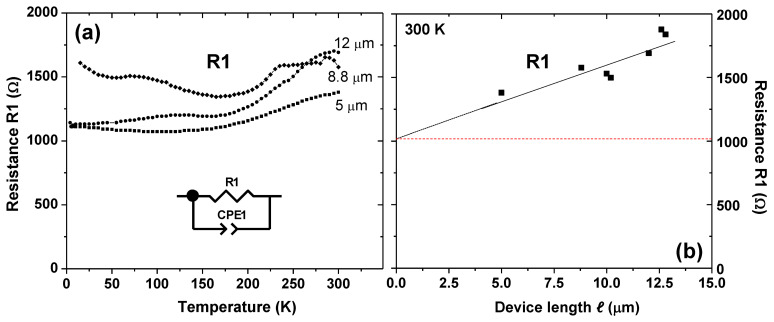
(**a**) *T*-dependence of resistance R1 from the models in Figure 3e and in the figure inset for devices with different length *ℓ* from sample B. (**b**) The resistance R1 scales with *ℓ*, but the scaling curve does not pass through the origin of the graph, and a residual resistance of extrinsic origin of ≈1 kΩ is indicated.

The situation is different for sample B that had been fabricated with the smaller device width of *w* = 10 μm and, therefore, displays a higher nominal graphene resistance. The larger nominal graphene resistance leads to mainly capacitive contributions in the impedance spectra as depicted in Figure 3e, which can be fitted with a single R1-CPE1 element (see inset of Figure 5a).

The *T*-dependences of the resistance extracted from resistor R1 are depicted in Figure 5a. Figure 5b shows that R1 scales with the device length *ℓ* to some extent, but the scaling curve does not pass through the origin of the graph. Instead, a residual resistance of ≈1 kΩ is indicated. The graphene sheet resistances for the different device lengths of *ℓ* = 5, 8.8 and 12 μm were calculated to be approximately 2.4, 1.4 and 1.25 kΩ/□, respectively. In contrast to the more conducting devices in the first sample A, the resistor R1 here yields sheet resistances that change with the device length *ℓ*. The term “sheet resistance” may be problematic here, because R1 contains the graphene resistance and additional extrinsic contributions. The “sheet resistance” for the larger devices (8.8 and 12 μm) approaches the value obtained from the first sample A (≈1 kΩ/□). These findings suggest that R1 in sample B may not consist of the contributions from the graphene layer only but contains multiple contributions: (i) extrinsic contributions from the electrode resistance and possibly the measurement cables, which do not scale with *ℓ*, and (ii) the intrinsic graphene layer resistance that scales with *ℓ*. This is plausible since the dielectric data from the second more resistive sample B could be fitted only with one single R-CPE element, although several contributions are expected to be present.

Therefore, it is not possible in this case to disentangle the different dielectric contributions, which is debited to the fact that only the onset of one semicircle was detected in −*Z*’’ vs. *Z*’ plots. Thus, in sample B, the resistance R1 in the equivalent circuit model depicted in the insets of Figure 3e and Figure 5a may contain all resistive contributions to the sample and thus, simply represent the DC resistance. In this case, the technique of EIS is of limited use, because the DC resistance may be extracted more easily by simple DC charge transport measurements. The rather unusual *T*-dependencies of R1 shown in Figure 5a may be explained by the fact that R1 contains several contributions that all may exhibit different *R*-*T* dependencies.

## 5. Discussion & Conclusions

It was demonstrated here that the extrinsic electrode resistance and the intrinsic graphene resistance of encapsulated graphene layers can be disentangled under certain conditions using EIS. The dielectric data vary qualitatively with the nominal graphene resistance (not the sheet resistance). In the case of sufficiently low graphene resistance, the dielectric spectra are dominated by inductive contributions, which allows for disentanglement of electrode and graphene resistance by the application of an adequate equivalent circuit model. Conversely, higher nominal graphene resistance leads to predominantly capacitive contributions, in which case the deconvolution is not feasible due to the experimental high frequency limit of the EIS apparatus of 1 MHz.

It can be concluded that EIS can be applied to graphene-based devices to achieve disentanglement of the electrode resistance from the intrinsic graphene layer resistance, in the case that the graphene resistance is sufficiently low. It is anticipated that the equivalent circuits proposed here in this work may be generally valid for EIS characterizations of graphene layer devices in future studies.

## Data Availability

The data presented in this study are available on request from the corresponding author. The data are not publicly available due to intellectual property rights applying to such data.
